# Application of Patient-Specific Computational Fluid Dynamics in Anomalous Aortic Origin of Coronary Artery: A Systematic Review

**DOI:** 10.3390/jcdd10090384

**Published:** 2023-09-06

**Authors:** Anselm W. Stark, Andreas A. Giannopoulos, Alexander Pugachev, Isaac Shiri, Andreas Haeberlin, Lorenz Räber, Dominik Obrist, Christoph Gräni

**Affiliations:** 1 Department of Cardiology, Inselspital, Bern University Hospital, University of Bern, 3010 Bern, Switzerland; anselm.stark@insel.ch (A.W.S.); isaac.shirilord@insel.ch (I.S.); andreas.haeberlin@insel.ch (A.H.); lorenz.raeber@insel.ch (L.R.); 2 Department of Nuclear Medicine, Cardiac Imaging, University Hospital Zurich, 8091 Zurich, Switzerland; andreas.giannopoulos@usz.ch; 3 Simq GmbH, 85567 Grafing bei München, Germany; apugachev@simq.de; 4 ARTORG Center for Biomedical Engineering Research, Faculty of Medicine, University of Bern, 3008 Bern, Switzerland; dominik.obrist@unibe.ch

**Keywords:** CFD, computational fluid dynamics, AAOCA, anomalous coronary artery

## Abstract

Anomalous aortic origin of a coronary artery (AAOCA) is a rare congenital heart condition with fixed and dynamic stenotic elements, potentially causing ischemia. Invasive coronary angiography under stress is the established method for assessing hemodynamics in AAOCA, yet it is costly, technically intricate, and uncomfortable. Computational fluid dynamics (CFD) simulations offer a noninvasive alternative for patient-specific hemodynamic analysis in AAOCA. This systematic review examines the role of CFD simulations in AAOCA, encompassing patient-specific modeling, noninvasive imaging-based boundary conditions, and flow characteristics. Screening articles using AAOCA and CFD-related terms prior to February 2023 yielded 19 publications, covering 370 patients. Over the past four years, 12 (63%) publications (259 patients) employed *dedicated CFD* models, whereas 7 (37%) publications (111 patients) used *general-purpose CFD* models. *Dedicated CFD* models were validated for fixed stenosis but lacked dynamic component representation. *General-purpose CFD* models exhibited variability and limitations, with fluid–solid interaction models showing promise. Interest in CFD modeling of AAOCA has surged recently, mainly utilizing dedicated models. However, these models inadequately replicate hemodynamics, necessitating novel CFD approaches to accurately simulate pathophysiological changes in AAOCA under stress conditions.

## 1. Introduction

**Figure 1:**
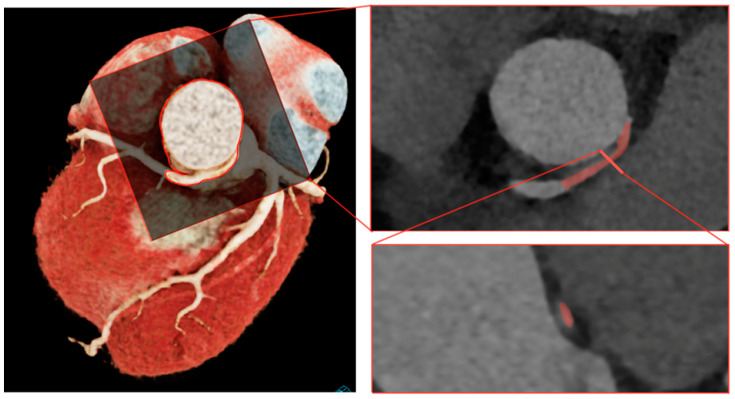
Anatomical high-risk features with an intramural course in red: an acute take-off angle (top right) and an elliptic vessel shape and proximal narrowing (bottom right).

Anomalous aortic origin of a coronary artery (AAOCA) is a rare congenital cardiac condition with a prevalence of approximately 1% in the general population [1]. Autopsy reports of sudden cardiac death in young athletes and military recruits proved that instances of AAOCA were in up to one third of the cases as the underlying cause [2,3,4,5]. However, the risk for patients living with AAOCA is likely much lower, as recently reported [6]. Furthermore, the issue of optimal management of AAOCA is becoming more important by the fact that rising utilization of cardiovascular imaging, particularly coronary computed tomography angiography (CCTA) and cardiac magnetic resonance imaging (CMR), leads to a greater absolute number of newly diagnosed AAOCA [1]. It is unclear to what extent patients have hemodynamically relevant AAOCA and how many only represent innocent bystanders and should be considered as incidental findings [7]. Therefore, adequate ischemia testing is of utmost importance, before moving towards management decisions. The variant of AAOCA with an interarterial course between the great arteries (i.e., aorta and pulmonary artery) is historically considered malignant due to an anticipated higher risk for sudden cardiac death [8,9]. However, it is widely accepted that the interarterial course per se is usually not the most important factor but rather acts as a surrogate marker for other anatomical high-risk features, including an acute take-off angle, slit-like ostium, elliptic vessel shape, proximal narrowing, and the most important high-risk feature of an intramural course (IMC) (see Figure 1).

These stenotic components can be divided into both fixed and dynamic components, whereby fixed stenosis is represented by a smaller and deformed ostial area, and dynamic stenosis is represented by the phasic lateral compression of the intramural segment throughout the cardiac cycle, which is aggravated with increasing aortic pressure, stroke volume, and heart rate during strenuous activity. The lateral compression of the IMC manifests mainly during systole and worsens with exercise, where also diastole is affected depending on the blood pressure. This has been verified in several studies, where the effect was quantified using intravascular ultrasound (IVUS) and measured fractional flow reserve (FFR) [10,11,12,13]. In fact, IVUS imaging showed a phasic reduction in the mean ostial area in systole in resting conditions, which aggravated under stress. FFR can be measured invasively using a pressure wire and represents the ratio of pressure distal to a coronary lesion and aortic pressure in resting conditions and under pharmacological testing. Different methods exist for pharmacological testing. In coronary artery disease (i.e., fixed stenosis), vasodilatation is usually used, such as adenosine or regadenosone. However, this method is not recommended in AAOCA, as the dynamic components would not be covered under vasodilatation. Although physical exercise would be preferred, it is usually not feasible in the catheter laboratory or in noninvasive imaging. Therefore, dobutamine, an inotropic/chronotropic agent, is a pharmacological alternative to mimic exercise conditions with increasing myocardial contraction, heart rate, and cardiac output. As a consequence, invasively measured FFR and IVUS are seen as the gold standard. However, this procedure is expensive, invasive, uncomfortable for the patient, associated with a non-negligible risk, and requires technical expertise from experienced interventional cardiologists. Therefore, virtual numerical simulation of stress conditions in AAOCA would be of great interest. Recent advances in computational fluid dynamics (CFD), a powerful noninvasive and versatile tool for analyzing fluid behavior in complex systems, have been used to study hemodynamics in AAOCA.

CFD for blood flow generally encompasses several sequential steps and employs various engineering tools. The process commences with the utilization of medical imaging data to construct a geometric representation. This geometry is subsequently transformed into a mesh structure. The discrete components of this mesh necessitate the assignment of distinct properties.

Within this context, the walls of blood vessels are ascribed either rigid or elastic characteristics, whereas blood itself can be modeled as a Newtonian or non-Newtonian fluid. In instances involving non-Newtonian fluids, precise models for blood viscosity must be implemented. Furthermore, the imposition of boundary conditions becomes paramount at the inlet and outlet locations. Following this, the Navier–Stokes equations—comprising a system of partial differential equations—can be addressed through dedicated software designed for solving the specific problem at hand [14,15].

However, it remains unclear how blood flow in the specific case of AAOCA should be modelled, as assumptions made for the boundary conditions and the 3D modelled geometry can strongly influence the results obtained. Furthermore, the spatial resolution of the underlying imaging modality used to construct the geometry is essential for accurately depicting reality and has the potential to significantly impact the validity of the results. Furthermore, how fixed and stenotic dynamic components should be modelled is not known.

This review aims to describe the current role of CFD models in patients with AAOCA; compare them based on imaging modality, underlying physiological models, and selected boundary conditions; and highlight strengths and limitations of the different simulation models.

## 2. Materials and Methods

A systematic search was conducted between December 2022 and February 2023 for all publications published prior to February 2023, related to CFD in AAOCA in the PubMed and EMBASE databases, using the following search terms: “computational fluid dynamics”, “fluid dynamics”, “CFD”, “flow simulation”, “wall shear stress”, “CT FFR”, “cFFR”, “FFR CT”, “quantitative flow ratio”, or “QFR” AND one of the following “coronary artery anomaly”, “AAOCA”, “ACAOS”, “coronary AND anomal*”, “anomalous AND coro*”, “coronary anomaly”, or “anomaly coronary vessel”. Medical Subject Headings (MeSH) terms were also included in the search. Papers were included if they studied AAOCA with an interarterial course and if CFD analysis was performed. Studies on other coronary anomalies, other coronary pathologies, analytical or experimental fluid dynamics, and solid mechanics finite element analysis were excluded. In addition, all reviews, posters, abstracts, or comments were excluded, and only original research papers and case reports were included. Of the included studies, all references were further screened if they met the inclusion criteria, and all eligible publications were included in the final analysis (cross-references).

The calculation of the solution for the Navier–Stokes equation of a given problem is usually solved with the use of a specific software called “solver”, rather than writing the code for the computational methods needed to solve the underlying differential equations. These solver software can further be divided into pre-assembled *dedicated CFD* models for coronary arteries or *general-purpose CFD* models (see Table 1). Dedicated CFD models are developed for one specific problem (e.g., coronary artery stenosis), and the model is then simplified as much as possible to save computational costs. General purpose CFD models allow one to change boundary conditions, temporal steps, complexity of the model, etc., while providing the necessary software to solve the underlying differential equations.

**Figure 2:**
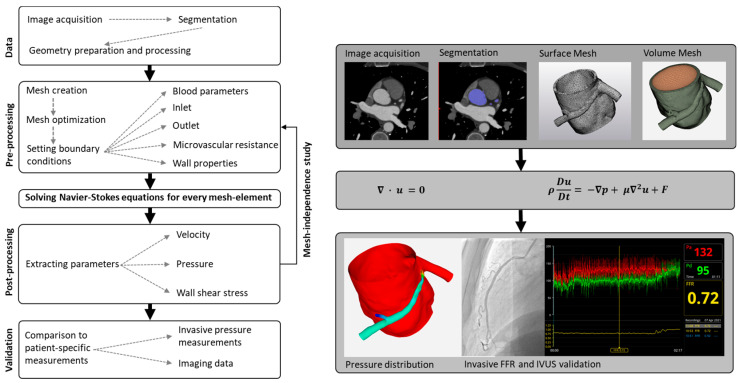
General workflow of computational fluid dynamics.

While *dedicated CFD* models use computational methods to analyze blood flow through the coronary arteries, they are not considered traditional CFD methods because they use simplified models with fixed assumptions specific for coronary arteries, instead of solving the full Navier–Stokes equations. For *dedicated CFD* models, it is unclear whether existing CFD models, which are established for CT-FFR or invasive angiography-derived quantitative flow ratio (QFR) in coronary artery disease (fixed stenosis), can be used in the same way for AAOCA (fixed and stenotic components), due to possible differences in boundary conditions. Studies with *general-purpose CFD* models were further analyzed according to the different steps within the workflow of CFD modelling (see Figure 2).

The main steps of the general workflow of computational fluid dynamics in AAOCA can be summarized as follows: data acquisition, pre-processing, solving, post-processing, and validation.

### 2.1. General Workflow of Computational Fluid Dynamics Modelling

Although the CFD workflow for AAOCA may vary, a general workflow can be defined for coronary arteries. The workflow typically involves the following steps:

#### 2.1.1. Step 1: Segmentation

The first necessary step begins with the reconstructing of the geometry of interest in a 3D space. For the CFD analysis of coronary arteries, a 3D model of the vessels is segmented from medical imaging data, most commonly CT scans. This process involves separating the aorta and coronary arteries from the surrounding tissues and creating a digital 3D model. The reconstructed 3D geometry usually requires further processing before it can be used in CFD analysis (e.g., extracting inlet and outlet boundaries and generating inflow and outflow regions).

#### 2.1.2. Step 2: Mesh Creation

The next step is to create a computational mesh or grid of small elements that discretize the geometry of the 3D model. This is necessary for solving the Navier–Stokes equations, which describe the fluid flow in the arteries. The mesh should be fine enough to capture the flow details but not so fine that it becomes computationally too expensive.

#### 2.1.3. Step 3: Setting Boundary Conditions

Boundary conditions define the blood’s physical properties and the artery’s geometry. Inlet and outlet boundary conditions specify the location and rate of blood flow into and out of the artery. Resistance boundary conditions describe the resistance to flow in the artery, and wall properties describe the mechanical properties of the artery wall.

#### 2.1.4. Step 4: Solve Navier–Stokes Equations

The Navier–Stokes equations are a system of partial differential equations describing fluid flow in the artery. In cases of incompressible fluid with constant dynamic viscosity, the Navier–Stokes equations can be expressed as follows [35]:

(1) Continuity Equation   ∇ · u=0,

(2) $$\mathrm{Momentum}\;\mathrm{Equations}\;\;\;\rho \frac{Du}{Dt}=-\nabla p+\mu \nabla^{2}u+F,$$

∇ · divergence

u  flow velocity

ρ  (mass) density

∇  Nabla operator

p  pressure

μ  dynamic viscosity

$\nabla^{2}$ LaPlace operator

F  gravitational forces

Solving these equations requires numerical methods, and the solution provides information on velocity, pressure, and other flow variables in the artery.

#### 2.1.5. Step 5: Post-Processing

Once the solution of the Navier–Stokes equations is completed, post-processing is performed to extract relevant information from the data. This can include calculating, e.g., the flow rate, pressure drop, and wall shear stress.

#### 2.1.6. Step 6: Validation against Clinical Data

Finally, the results of the CFD simulation are compared to clinical data to validate the accuracy of the simulation. This involves comparing the calculated flow variables to measured values in the corresponding patients to ensure that the simulation accurately captures the flow behavior in the artery.

### 2.2. Statistics

Comparable variables between studies were expressed as a weighted average with median and interquartile range. Wilcoxon tests were conducted for case reports in which CT-FFR and FFR_adenosine_ were available. All statistical analyses were performed using R Studio (v4.2.3).

## 3. Results

**Figure 3:**
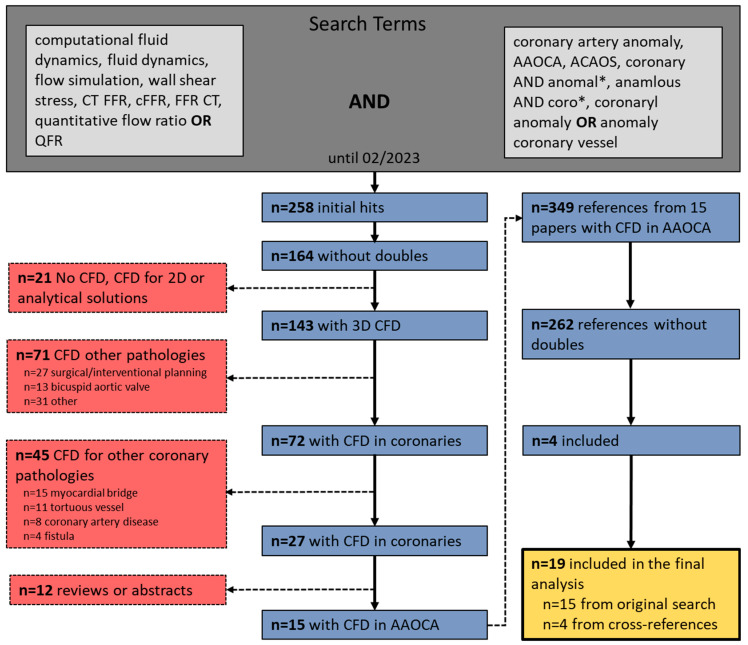
Consort flow of the study selection process. Abbreviations: CFD = computational fluid dynamics, CT FFR = computed tomography-derived fractional flow reserve, cFFR = CT-FFR from Siemens Healthineers, QFR = quantitative flow ratio, AAOCA = anomalous aortic origin of a coronary artery, ACAOS = anomalous coronary artery from the opposite sinus of Valsalva and * = wildcard symbol, that broadens a search by finding words that start with the same letters

A total of 258 studies were initially identified using the key terms. Out of them, 15 studies fulfilled the final inclusion criteria, and an additional 4 studies were identified through cross-reference searches, resulting in a total of 19 papers published until February 2023 for the final analysis (see Figure 3). A total of 19 publications, including 370 patients, mostly published within the last 4 years, were identified and included in the final review. Therefore, in 12 (63%) studies (i.e., 259, 70% patients), a *dedicated CFD* model was used, whereas *general-purpose CFD* models were applied in 7 (37%) reports (i.e., 111, 30% patients).

### 3.1. Dedicated CFD Coronary Artery Software

Out of 12 *dedicated CFD* models, 3 employed cFFR by Siemens Healthineers, comprising a total of 96 patients; 8 studies used FFR_CT_ by HeartFlow^®^ (Redwood City, CA, USA), comprising a total of 122 patients; and 1 used QFR by Medis, comprising a total of 41 patients. The weighted mean age of these 259 patients was 59.3 ± 13.8 years. Of these patients, CT-FFR was performed in 135 cases with an AAOCA and an additional interarterial course. The study by Ferrag et al. [24] was excluded in the weighted analysis, as they used a subgroup of the same database as Adjedj et al. [16]. In the study by Adjedj et al. [16,23], only 33 patients (61%) had an interarterial course, including 2 with L-AAOCA and 31 with R-AAOCA. The weighted mean FFR of these patients was 0.91, with a weighted mean FFR of 0.91 (n = 131) for R-AAOCA and 0.84 (n = 4) for L-AAOCA.

Tang et al. [22] studied 98 patients with an interarterial R-AAOCA who underwent CCTA, and CT-FFR could be performed in 94 patients (96%). They analyzed CT-FFR and the following anatomical high-risk features: proximal vessel morphology (oval or slit-like), take-off angle, take-off level (below or above the pulmonary valve), take-off type, IMC, % proximal narrowing area, length of narrowing, minimum luminal area (MLA) at systole and diastole, and vessel compression index [22]. They further investigated the association between CT-FFR and interarterial R-AAOCA and performed receiver operating characteristic (ROC) analysis of CT-FFR < 0.8 to detect an interarterial course in R-AAOCA. They found that patients had significantly more typical angina (29.4% vs. 7.8%) and atypical angina (29.4% vs. 6.5%) in the group with CT-FFR < 0.8 (n = 17) than in the group with CT-FFR > 0.8 (n = 77). Slit-like ostium (88.2% vs. 23.4%) and IMC (94.1% vs. 31.2%) were also more frequently present. MLA was lower in diastole (3.8 mm^2^ vs. 5.3 mm^2^) but not in systole (4.8 mm^2^ vs. 5.9 mm^2^). The best model to predict CT-FFR < 0.8 included IMC, take-off level above the pulmonary valve, and slit-like ostium, with an area under the curve (AUC) of 0.92.

Ferrag et al. [24] included 106 patients with AAOCA, of whom CT-FFR could be performed in 62 patients (58%). Patients in whom CT-FFR could not be performed were due to impaired image quality and software technical issues. They analyzed anatomical high-risk features such as luminal surface narrowing, luminal eccentricity, take-off angle, and IMC in addition to CT-FFR analysis. They then evaluated the performance of these high-risk features in predicting distal CT-FFR < 0.8. Among the included patients (i.e., 11 L-AAOCA, 37 R-AAOCA, and 14 AAOCA of a circumflex), 37 patients showed an interarterial course. Surface narrowing >50% had an odds ratio (OR) of 4.5 (95% confidence interval [95%CI] of 1.3–13.7), eccentricity degree >1.5 had an OR of 2.6 (95%CI 0.8–8.4), angle <30° had an OR of 3.5 (95%CI 1.1–11.9), and IMC had an OR of 9.2 (95%CI 1.7–49.9) to predict CT-FFR < 0.8. Additionally, they used ROC analysis to predict IMC using CT-FFR, which resulted in an AUC of 0.89.

Adjedj et al. [23] included 105 patients, of whom CT-FFR could be performed in 54 (51%) patients (i.e., 8 L-AAOCA, 32 R-AAOCA, and 16 AAOCA of a circumflex), with 31 patients having an interarterial course. Patients with an interarterial course did not present differently from patients without an interarterial course in terms of symptoms, noninvasive stress testing, or CT-FFR values (0.90 ± 0.10 vs. 0.91 ± 0.09, and *p* = 0.516).

QFR from invasive angiograms was assessed by Adjedj et al. [27] in 128 patients, and QFR could be performed in 41 of these patients (32%). The mean stenosis percentage was 17.8 ± 16.3%, and the mean QFR was 0.9 ± 0.1. A sub-analysis of patients with visually assessed stenosis <50% vs. >50% showed values of 0.90 ± 0.1 vs. 0.88 ± 0.09 (*p* = 0.27).

**Figure 4:**
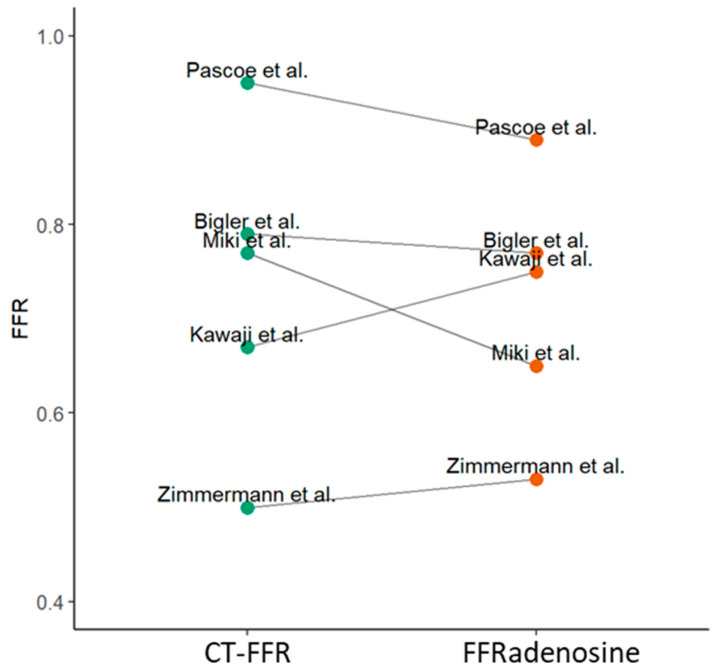
Comparison of computed tomography-derived fractional flow reserve (CT-FFR) against invasively measured FFR adenosine in case reports where this information was available. Comparison was performed by a paired Wilcoxon signed rank test. There was no significant difference between CT-FFR and FFR adenosine (*p* = 0.81). Case reports used for this analysis: Pascoe 2019 [21], Bigler 2022 [26], Miki 2018 [19], Kawaji 2016 [17] and Zimmermann 2017 [18]

The case reports focused on the use of CT-FFR for decision-making in surgery. In five of the case reports, invasively measured FFR under adenosine was available for comparison (see Table 2). There was no difference between CT-FFR and FFR adenosine (*p* = 0.81), and the mean absolute error of CT-FFR compared to FFR adenosine was 0.06 (see Figure 4).

### 3.2. General-Purpose CFD Models

A total of seven papers were identified using *general-purpose CFD* models, comprising 111 patients with a weighted mean age of 43.4 years, with only Razavi et al. [32] including children with a median age of 13.5 years (range: 9–17 years). The papers were subsequently compared for the various steps of the workflow proposed in the introduction. A summary of these findings is presented in Table 3.

#### 3.2.1. Image Acquisition and Segmentation

Except for the study by Razavi et al. [32] who used CMR, all other groups used CCTA images for segmentation. Segmentation approaches varied between studies, with most employing a semi-automated method with consequent manual correction. In six studies, the aortic root and coronaries were segmented together, whereas only the coronaries were segmented in one study. Post-processing details, such as geometry smoothing, were not consistently reported, except for the used software. Jiang et al. [34], who incorporated wall thickness in addition to lumen measurements, reported extrusion parameters for aortic and coronary wall thickness (1.7 mm and 0.9 mm).

#### 3.2.2. Mesh Generation

The approaches to mesh creation were quite heterogeneous, with varying mesh sizes and expansion layers. Mesh independence studies were performed by four teams. Optimal mesh sizes were reported as 0.8 mm for the fluid domain and 1 mm for the vessel wall in the model by Cong et al. [29,31], 0.02 mm for the fluid domain in the model by Chidyagwai et al. [33], and an optimum of 3.5 e6 elements for the fluid domain in the model by Razavi et al. [32]. Jiang et al. [34] did not report the exact number for the mesh size but performed a mesh independence study and reported that the wall was always represented by at least two elements in diameter and the fluid domain by five.

#### 3.2.3. Blood Model

Blood density was reported in six out of seven papers and set to 1060 kg/m^3^ in five and 1040 kg/m^3^ in one case. Blood was assumed as Newtonian in five cases. Viscosity was set to 0.04 dyne s/cm^2^ when reported. A non-Newtonian Carreau fluid model was applied in two cases [36].

#### 3.2.4. Boundary Conditions at the Inlet

Inlet boundary conditions varied across studies and were dependent on whether a pulsatile or transient model was used. Cong et al. [31] employed a pulsatile flow generated by a Fourier series to match a standard aortic pressure curve of a patient. Rigatelli et al. [28] used diastolic pressure values from ergometric stress tests of healthy athletes as pressure inlets for both of their studies. In another paper by Cong et al. [29], a steady flow was assumed with a constant velocity of 1 m/s for the aorta. Chidyagwai et al. [33] used a pulsatile velocity profile derived from echocardiographic Doppler measurements for LCA and RCA inflows. The cardiac output was assumed to be three times higher for flow during stress conditions. Razavi et al. [32] used the volumetric flow derived from the CMR exam and then divided the flow into the different branches using Murray’s law.

Jiang et al. [34] used the pressure waveform from the instantaneous wave-free ratio (iFR) measurements at rest and stress. The iFR represents the ratio between the distal and proximal coronary pressure during a specific period in the cardiac cycle called the wave-free period, which is a phase of minimal flow-related pressure changes. IFR measurements require no adenosine compared to fractional flow reserve, which is assessed with adenosine. The ratio of the systolic to the diastolic phase was further adjusted to heart rate, with increasing length of the systolic phase using the following approximation, based on the echo-derived linear model of heart rate and systolic period:

(3) $$t_{systole}\left[ms\right]=425\left[ms\right]-1.5\left[ms\times min\right]\times heart\;rate\left[\frac{1}{min}\right],$$

heart rate in beats per minute

$t_{systole}$ time until systole in ms

#### 3.2.5. Boundary Conditions at the Outlet

Rigatelli et al. [28,30] did not provide information about their outflow boundary conditions. Three papers utilized [32,33,34] a lumped parameter model (LPM) to simulate the microcirculation, which included resistance, capacitance, and inductance. Chidyagwai et al. [33] used a LPM to model the microvascular resistance in order to match clinical diastolic and systolic pressure values. The number of outlets varied between 2 and 10 for the left coronary arteries, with corresponding resistance values ranging from 0.894 to 50.8 mmHg s/m^3^. For the right coronary artery, the number of outlets varied from 1 to 3, with resistance values ranging from 0.698 to 8.23 mmHg s/m^3^. Razavi et al. [32] used a LPM with resistance values adjusted to match 4% of the total cardiac output. The exact set-up of the LPM was not described, but it included coronary resistance parameters for arterial, micro-arterial, and venous resistance and capacitance for arterial and intramyocardial components. They also modeled the aortic Windkessel effect with a proximal resistance, capacitance, and distal resistance. Jiang et al. [34] also used a LPM and assigned 4–5% of the total cardiac output to the coronary arteries. They further assigned 30% of the coronary flow to the right coronary artery based on Doppler measurements and estimates of myocardial mass perfused by each coronary. Cong et al. used an approach in which they set the outlet as a constant pressure of 56 Pa for the aorta and 0 Pa for the coronaries in their first study [31], 93 mmHg for the aorta, 81.83 mmHg for the left coronary artery, and 92.7 mmHg for the right coronary artery in their second study [29].

#### 3.2.6. Modelling of the Aortic and Coronary Walls

Rigatelli et al. [28,30] did not provide any information regarding the wall parameters. Cong et al. [31] employed a no-slip condition and assumed a linearly elastic wall with a wall material density of 1150 kg/m^3^, Young’s modulus of 5 MPa, and a Poisson’s ratio of 0.45. Chidyagwai et al. [33] used a rigid wall model with a no-slip condition. Jiang et al. [34] utilized a linearly elastic wall material for both the aorta and coronary arteries with a density of 1200 kg/m^3,^ a Young’s modulus of 1.5 MPa, and a Poisson ratio of 0.49.

#### 3.2.7. Post-Processing

Cong et al. [31] compared the wall shear stress (WSS), pressure, and volumetric flow in R-AAOCA (n = 26) to normal RCA (n = 16) and found that the volumetric flow was significantly lower in the R-AAOCA group compared to normal RCA (1.14 ± 0.57 mL/s versus 3.5 ± 0.57 mL/s, *p* < 0.001). There was no difference in pressure between the two groups (12861 ± 80.5 Pa versus 12532 ± 80.5 Pa, *p* > 0.05) and no difference in WSS (1.73 ± 0.56 Pa versus 2.53 ± 0.56 Pa).

The same group [29] focused on comparing WSS and pressure between R-AAOCA (n = 26, mean age 62 ± 10.9 years) and normal RCA (n = 16, mean age 58 ± 8.7 years), as well as virtual patients with differing acute take-off angles. These parameters were then plotted over a cardiac cycle and compared at different time points. They found that pressure was significantly lower only during the time period of 1.32–1.46 s (R-AAOCA range [−72.5–256.5 Pa] versus normal range [28.1–411.9 Pa], and *p* < 0.001), and the volumetric flow was not significantly lower. WSS was slightly higher during the time period of 1.24–1.28 s.

Rigatelli et al. conducted two studies [28,30] on patients with L-AAOCA and an intramural course (IMC). In their first study, they compared WSS and vorticity magnitude (VM) between L-AAOCA patients with (n = 6) and without (n = 7) IMC, with a mean age of 45.1 ± 8.2 years. While they did not find a significant difference in pressure during rest and exercise (1.10e4 vs. 1.10e4, *p* = 0.8), they observed a significant increase in WSS and VM in L-AAOCA patients with an IMC during exercise, whereas this effect was negligible in L-AAOCA patients without an IMC. In their second study, Rigatelli et al. [30] focused on virtual stenting of IMC in L-AAOCA and R-AAOCA in 21 symptomatic patients with AAOCA, with a mean age of 46.1 ± 8.1 years (L-AAOCA n = 9, R-AAOCA n = 12). They found that virtual stenting resulted in a significant decrease in WSS values in the IMC, leading to normal values (9.5 ± 0.2 Pa (L-AAOCA) and 8.6 ± 0.2 Pa (R-AAOCA) versus 8.6 ± 0.2 Pa (L-AAOCA) and 6.8 ± 0.1 Pa (R-AAOCA), and *p* < 0.001). Furthermore, they observed a significant reduction in the axial twist of the IMC after virtual stenting.

Chidyagwai et al. [33] conducted a case–control study with 13 patients (L-AAOCA n = 2, R-AAOCA n = 6, and controls n = 5) and a median age of 13, focusing on WSS and oscillatory shear index in L-AAOCA and R-AAOCA patients during rest and exercise compared to normal reference vessels. They found that both groups displayed higher mean WSS values compared to normal coronary arteries. The WSS distribution in the IMC was skewed towards higher values. However, the oscillatory shear index (OSI) did not differ in AAOCA patients compared to normal coronaries. WSS was assessed before and after unroofing in one patient, and a significant reduction was found (5.74 ± 1.32 vs. 1.47 ± 1.01 Pa, *p* = 0.011). No significant change was observed for the OSI before and after the procedure.

Razavi et al. [32] focused on L-AAOCA and R-AAOCA patients before and after the unroofing procedure. They included six patients with a median age of 13.5 [9,10,11,12,13,14,15,16,17] years. WSS significantly decreased after unroofing in the IMC and after the IMC. The velocity profile also changed from before to after surgery, where the profile was skewed towards the outer wall of the intramural course in preoperative cases, reaching maximum velocity values of 190 cm/s. Comparison of L-AAOCA to R-AAOCA showed that L-AAOCA had higher WSS values, and post-unroofing showed similar trends in both groups. An analysis of OSI showed no differences between normal coronaries and AAOCA or between pre- and post-unroofing cases.

Jiang et al. [34] enrolled 6 patients with R-AAOCA and a mean age of 48 ± 14 years. The study aimed to compare invasively measured iFR to simulated iFR during rest and dobutamine stress. They observed a decrease in the invasively measured iFR values from 0.96 ± 0.02 to 0.87 ± 0.06. For resting and stress conditions, the simulated iFRs had root mean squared errors (RMSEs) of 0.02 and 0.05, respectively, compared to the invasively measured iFRs. Bland–Altman analysis demonstrated the agreement between the measured and simulated iFR values within differences ≤0.05 and one outlier with 0.09. Additionally, the authors included a pulmonary artery in their analysis to model the suggested scissor-like mechanism and found that iFR results changed by less than 1%.

## 4. Discussion

The results of the current systematic review can be summarized as follows: The use of CFD in the setting of AAOCA has gained much attention in recent years. All studies were retrospective and yet, there is in general limited data available and approaches differed across studies. Most of the studies used CCTA data and focused on dedicated CFD software validated against fixed stenosis of coronary artery disease, such as CT-FFR and QFR from invasive angiograms and might therefore, not entirely cover the spectrum of pathophysiological changes. Most studies used CFD to predict pathological flow patterns and guide decision-making for revascularization or assess changes in flow patterns after surgery. The patient population consisted mainly of middle-aged patients, with only two studies including children.

### 4.1. Dedicated CFD Models

The goal of CFD analysis is to accurately capture the real-world behavior of the studied system while remaining computationally efficient. This objective is exemplified by the emergence of commercial software such as FFR_CT_ by HeartFlow^®^, which is FDA-approved, can predict FFR from CTTA, and has already been linked to outcomes in several trials [37,38,39,40]. Another alternative for CT-derived FFR is cFFR (a research software) by Siemens Healthineers, which can be performed on a local server with significantly faster computation times (5–10 min compared to 8 h) [41]. Angiography-derived FFR calculation can also be achieved using the QFR tool by Medis QFR (Medis Medical Imaging System, Leiden, Netherlands), which has shown favorable outcomes and comparisons to invasively measured FFR in several papers[42,43,44,45]. The commercial success of these tools demonstrates the growing interest in applying CFD to clinical problems.

However, as *dedicated CFD* software aims to reduce computational cost as much as possible, they use a simplified CFD model, which is focused on a fixed stenotic component, like coronary artery disease, which differs pathophysiologically from AAOCA. For example, FFR_CT_ by HeartFlow^®^ employs a transient state simulation with blood modelled as a Newtonian fluid and a lumped parameter model for microvascular resistance. The walls are assumed to be rigid, and FFR is reported for diastole with minimal resistance to mimic the effect of adenosine [46,47,48]. Similarly, cFFR by Siemens Healthineers uses a transient flow and blood modelled as a Newtonian fluid. The microvascular resistance is minimized to mimic the effect of adenosine, and no information about wall properties is available [41].

However, in AAOCA, there is most often an additional dynamic component present, as suggested by Bigler et al. [49], which is not considered in available *dedicated CFD* software. Therefore, the results of *dedicated CFD* software are likely to underestimate FFR during exercise conditions depicted by FFR_dobutamine_. This was demonstrated in the case report by Bigler et al. [26] of a patient with an R-ACAOS, where CT-FFR was 0.79, FFR_adenosine_ was 0.77, and FFR_dobutamine_ was 0.72. With resting aortic pressures, lateral compression is probably negligible, and FFR is mainly influenced by fixed stenotic components, such as the ostial area reduction, due to a slit-like ostium and an elliptic vessel shape. This would match the findings that in five case reports the invasively measured FFR_adenosine_ was available and showed no significant difference to CT-FFR values. However, as the risk for sudden cardiac death has been described, mainly in the setting of strenuous exercise, CT-FFR might provide a false sense of security. In line, Lee et al. [13] showed in their study, including 37 patients, a change from FFR_adenosine_ of 0.91 ± 0.06 to FFR_dobutamine_ of 0.89 ± 0.06. On the contrary, they observed a discrepancy, in which the FFR_dobutamine_ was unexpectedly lower in three patients. Yet, the exact contribution of the dynamic lateral compression to ischemia is still not well understood. [50] Other case reports have reported the same findings, where FFR_dobutamine_ was lower [26,34,51]. Nevertheless, CT-FFR should be validated against the gold-standard FFR_dobutamine_ in order to be a sensitive test assessing fixed and dynamic components. However, current CT-FFR tools could still be useful, not sensitive, but initially specific tools (pathological CT-FFR is truly pathological). Tang et al. and Ferrag et al. [22,24] both reported that high-risk anatomical features were predictive of CT-FFR values below 0.8, indicating a correlation between anatomy and CT-FFR. Nevertheless, the studies by Ferrag et al. [24] and Adjedj et al. [16,23] both had a considerable number of unanalyzable patients, which could be a restricting factor for using this tool. In this context, it is unclear whether the newly developed photon-counting CT, with its improved spatial resolution, could enhance the applicability of CT-FFR and reduce the number of unanalyzable scans.

QFR was only assessed by one group, and it did not show promising results, especially since it could not be assessed in most patients [27]. This may be due to the fact that the stenotic segment of AAOCA lies intramurally and is elliptically shaped, making it difficult to visualize from only two different angles from invasive angiography, which would have to be perfectly aligned. Additionally, there is significantly more overlap of structures due to the aortic root, which adds complexity to the analysis.

### 4.2. General-Purpose CFD Models

#### 4.2.1. Image Acquisition and Segmentation

**Figure 5:**
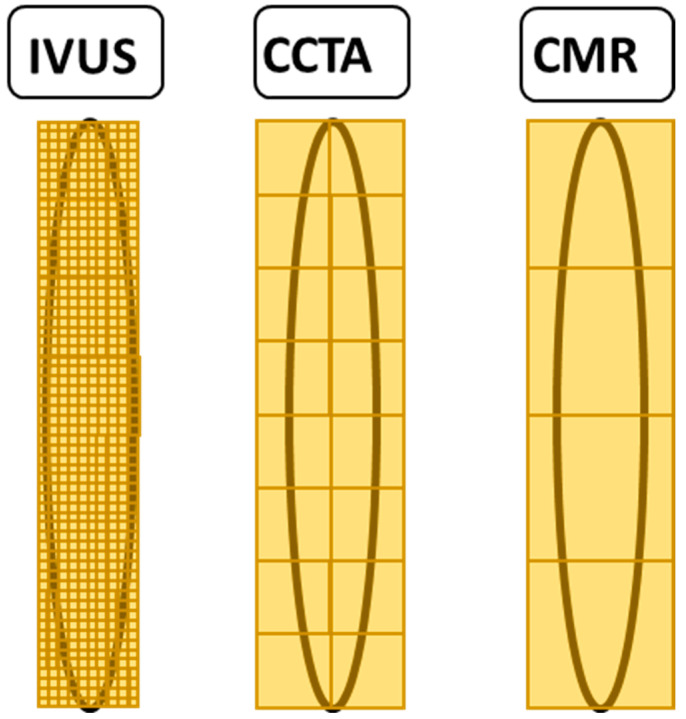
Comparing spatial resolution of the proximal intramural segment in AAOCA.

As the IMC of AAOCA typically has a smaller ostial area than normal coronary arteries [52], an imaging modality with the highest spatial resolution should be preferred to depict the IMC accurately. Most authors agree that CCTA is the preferred modality, although Razavi et al. [32] used CMR due to radiation issues for imaging in their pediatric population. While CMR has a spatial resolution of 1.5 × 1.5 mm in plane at best and 5 mm through-plane, CT has a standard resolution of 0.75 × 0.75 mm, and novel Photon-Counting CT scanner will offer from 0.3 mm up to 0.1 mm spatial resolution [53,54,55]. However, IVUS and optical coherence tomography (OCT) offer even higher resolutions of 0.1 × 0.1 mm and 0.01 × 0.01 mm, respectively, depending on the specific machine and catheter used (Figure 5), with higher temporal resolution compared to CT [56].

The comparison of the spatial resolution of different imaging modalities of the intramural segment with height 6 mm and width 0.9 mm are depicted. Cardiovascular magnetic resonance imaging (CMR) has a resolution of 1.5 × 1.5 mm, coronary computed tomography angiography (CCTA) has 0.75 × 0.75 mm, and intravascular ultrasound (IVUS) has a resolution of 0.01 × 0.01 mm.

Whereas the typical diameter of a coronary artery is around 3–4 mm, the intramural segment of AAOCA is typically smaller. With a slice thickness of 0.7 mm in CCTA, the limited image resolution can result in a measurement error of up to 20 percent, significantly affecting the overall findings. A weak correlation with IVUS images and low ICC values for CCTA has been described before and could therefore greatly influence the results of the simulations [57]. However, how novel Photon Counting CCTA images with an ultra-high resolution of up to 0.1/0.2 mm impact on future CFD models needs to be evaluated. While IVUS and OCT can accurately depict the IMC, they are invasive and cannot provide 3D structural information of the coronary tree. Nonetheless, they could serve as the gold standard to compare CCTA segmentation or improve geometric precision with the fusion of IVUS/OCT images with CCTA [58]. In addition to the resolution issue, the reproducibility of different models generated from different imaging modalities, particularly for CCTA, has to be taken into account. In a recent study by Ferraro et al. [59], the reproducibility for the ostial area analysis showed an intra-reader reliability of 0.83 and an inter-reader reliability of 0.75. Another important factor that can influence geometry is the applied post-processing, such as smoothing and surface mesh size. Attaining an optimal balance between convergence behavior and a realistic model that accurately represents the patient’s anatomy necessitates a meticulous contemplation of these factors.

#### 4.2.2. Mesh Generation

A mesh independence study was conducted by four out of the seven publications. Through the implementation of these studies, there is potential to substantially diminish computational expenditures while still achieving equivalent outcomes, with only a marginal increase in the required effort and should be promoted to become a standard procedure. How different geometric configurations for mesh elements have the potential to enhance convergence characteristics in the precise setting of AAOCA is unknown. Nevertheless, it is possible that the dimensions of the foundational surface geometry exert a greater influence on the results than the mesh elements that are utilized to depict it. For the same reason, the engineering tools to create the mesh should be reported due to their differing algorithms, and no information about mesh-quality assessment was provided.

#### 4.2.3. Blood Rheology

Despite blood being a non-Newtonian fluid, five of the seven groups adopted the simplifying assumption of treating blood as a Newtonian fluid in their investigations. In fact, previous studies suggested that blood can be assumed as a Newtonian fluid with a constant viscosity independent of shear rate for laminar flow in epicardial arteries [60,61,62]. However, in a recent study by Thondapu et al. [63], non-Newtonian WSS was compared to Newtonian WSS, and the former was found to estimate significantly higher time-averaged WSS and WSS gradients, potentially improving the accuracy of WSS measurements. If the fluid is assumed to be non-Newtonian, it is further unclear which model for viscosity is best suited. Other models than the Carreau model adopted by Rigatelli et al., for example, the Quemada model, have been successfully applied for the simulation of blood flow [15]. Furthermore, and mainly given the acute take-off angle and the IMC anatomy in AAOCA, whereby blood flow might not necessarily remain laminar, it remains unclear if the flow can be assumed as Newtonian. The potential advantages of employing non-Newtonian methodologies in achieving higher precision are counterbalanced by the concomitant escalation in computational expenses, and consensus is lacking on the blood models that are employed.

#### 4.2.4. Boundary Conditions of the Inlet and Outlet, Vessel Wall Properties

Inlet boundary conditions for AAOCA consider not only the coronary system but also the interplay between the aortic and coronary system. Patient-specific available clinical measurements include blood pressure, heart rate, echocardiography-derived parameters, and a lateral compression model, which is mainly observed during exercise with increased cardiac output. Rigid walls are inadequate to capture lateral compression. Modelling the microvascular system of the heart is challenging, and a lumped parameter model is commonly used. Jiang et al. [34] proposed the most realistic boundary conditions for AAOCA models, particularly as they were the only ones to consider lateral compression. However, their study population was small, and whether their results can predict hemodynamic relevance in a clinical setting is unclear.

To truly simulate the phenomenon of lateral compression, a computational fluid–solid interaction (FSI) model would most probably represent the ideal approach. These models are characterized by high computational costs and complexity, as they must accurately represent the deformation of the vessel’s intramural segment in addition to predicting the flow of the fluid. This entails making additional assumptions regarding the material properties of the aorta and coronary arteries. To improve the model’s accuracy, future studies should initially validate the structural model against IVUS or OCT during dobutamine stress, followed by modeling the flow within the generated mesh. For boundary conditions, all available invasively measured parameters as possible should be used to inform the CFD model and gradually replace them with noninvasive parameters to assess their effects on the outcomes.

#### 4.2.5. Validation

The selection of optimal boundary conditions is a key determinant of the accuracy and realism of the results obtained. Therefore, validating the models against in vivo acquired hemodynamic gold-standard dobutamine measurements is crucial. Although the validation step of newly developed models is critical, only a few studies have done so, and the majority have used FFR_adenosine_ as the reference standard, with only one case report, and one study validated their findings against dobutamine measurements. Jiang et al. [34] used iFR_dobutamine_, (i.e., iFR is a parameter originally proposed to be an adenosine free alternative to FFR). The index is calculated as a ratio of resting distal coronary pressure and resting aortic pressure, during a specific period in late diastole, called the wave-free period. In this period, intracoronary resistance is proposed to be constant and minimal. However, there are a lack of experimental data, and some studies have shown that both iFR and resistance decrease during the wave-free period [64,65]. The IDEAL study even proposed that resistance in the wave-free period is higher than in the whole-cycle hyperemic resistance [66]. McCray et al. were the first that proposed iFR_dobutamine_ for the assessment of R-AAOCA[67]; they did this based on an older study by Escaned et al., where they found that in myocardial bridging FFR_dobutamine_ for only the diastole represented a more precise marker to detect hemodynamic relevance [68]. Whether iFR or FFR_dobutamine_ differs or which one provides benefits are topics of interest for future research. Most importantly, in their model, the residual error during exercise was around 0.05, which was still a significant error, considering the narrow range that iFR values can take, especially for the normal range between 0.8 and 1.0.

Another problem was the non-physiologic values acquired by many of the other groups. Cong et al. [29] found normal values inside the coronary arteries with around 13,000 Pa, which corresponded to approximately 98 mmHg. However, they also found normal pressure values inside of the anomalous coronary, which were either due to anomalies with other anatomic high-risk features to reduce the luminal area or an unknown error within their generated model. In their second study [31], they acquired negative values during a large period of the cardiac cycle, which was non-physiological. Razavi et al. [32] reported a maximal velocity of 190 cm/s, which was extremely high compared to values that were reported for normal coronary arteries [69,70,71]. Other authors focused on wall-shear stress only, which is a value that is not possible to validate in vivo. While these values could provide additional information about hemodynamics in these patients, they should be reported together with other values that can be validated against patient data.

Following validation of the CFD models with invasive measurements, clinical validation with outcomes should be pursued, especially for parameters that cannot be directly measured, such as WSS.

### 4.3. Model Complexity

*Dedicated CFD* models, especially CT-FFR by Siemens Healthineers, aim to reduce the model’s complexity to reduce the computational time. The CFD analysis should always aim at reducing complexity while remaining accurate enough to depict the underlying flow phenomena. In the same manner, the *general-purpose CFD* models can be ordered by complexity. The simplest CFD models are 0D and consist of a lumped parameter model. While no studies used a 0D model alone, they were integrated into several of the more complex models. Three groups coupled a lumped parameter model to the outlet of the 3D model and set the boundary conditions through the lumped parameter model. One-dimensional models, which would also allow for the modelling of wave propagation along the vessel, were not used in any model. However, it has been suggested that these models are more important in the great vessels such as the aorta [72]. Two-dimensional models are probably not suited in the case of AAOCA since the intramural course represents an asymmetric stenosis with an elliptic vessel shape. It is, therefore, hard to accurately depict the stenosis degree in 2D.

The group of more complex models consists of 3D models: one-way and two-way FSI models. Three-dimensional models, in this case, are simulations that solve the Navier–Stokes equation over a 3D model that retains its original shape. In an FSI model, the 3D shape is modified by the fluid. In reality, the flow field is further modified by the changing solid, which then again creates a feedback loop on the solid until an equilibrium is achieved. A two-way FSI model depicts this feedback, whereas a one-way FSI model does not.

All studies created models with at least this level of complexity. Results were not uniformly reported, which makes the comparison between them harder. Jiang et al. [34] were the only ones to use an FSI model, which also showed aortic deformation and was unique in their approach. In their approach, they used a two-way FSI model, which, however, still had clinically relevant errors, especially under dobutamine stress with RSME 0.05. The FSI model of the vessel wall was described rather generally, and detailed information about parameters was not provided. The deformation of the model, and especially the lateral compression of the intramural segment of AAOCA, were in none of the studies validated against invasive measurements (i.e., IVUS). Cong et al. [29,31] used an FSI model for the coronary artery walls in their models compared to Razavi and Chidyagwai et al. [32,33]. However, the comparison is not feasible since one team focused on WSS stress in R-AAOCA and the other on L-AAOCA. This would be a topic of interest to see how the results of increasingly complex models differ from each other.

### 4.4. Technical Considerations

Traditional CFD focuses on solving the Navier–Stokes equations, which describe macroscopic flow, with different approaches such as the finite difference or finite volume method. However, there are also different methods to solve fluid flow such as the more recent Lattice Boltzmann method. This method focuses on solving microscopic flow to calculate macroscopic flow phenomena by focusing on a different set of differential equations. The main advantage of this method is the potential computational speedup through more efficient parallelization; this is especially important for complex simulations such as FSI models. However, the chosen method has also an influence of the acquired results [73]. Which method is best suited in the specific setting of AAOCA has to be evaluated in future studies. On the same note, GPU parallelization could be a potential factor to additionally speed up simulations, and it has already showed potential for the simulation of FFR [74].

### 4.5. Limitations

This review has several limitations; first, only a few studies were available for CFD in AAOCA, and most of these studies showed various approaches for CFD analysis, especially for the newly developed CFD models. Therefore, a systematic meta-analysis was not possible. Second, invasive measurements (i.e., FFR_adenosine_ and FFR_dobutamine_) were only available in a small part of the studies, limiting the validity of the results’ applicability.

## 5. Conclusions

Application of CFD in patients with an AAOCA is an emerging method that could unravel hemodynamic relevance and guide therapeutic management without the need of invasive diagnostics. However, contemporary *dedicated* and *general-purpose CFD* models seem to be so far unable to depict the entire spectrum of pathophysiological consequences of AAOCA, especially with regard to the dynamic components. Therefore, novel CFD approaches are needed in the context of AAOCA; employing computationally intensive methods may still be a viable option, as time constraints are not critical. The availability of a comprehensive dataset with invasively measured parameters from a sufficiently large sample size for validation is indispensable in this process.

## Figures and Tables

**Table 1:** Overview of the studies included in the final analysis.

First Author	Year	n	Type	Solver	Setting (R-AAOCA, L-AAOCA, Both)	Aim	Validation with Invasive Measurements
Adjedj et al. [16]	2016	1	Case Report	FFR_CT_ *	Left	CT-FFR for decision making	N/A
Kawaji et al. [17]	2017	1	Case Report	FFR_CT_ *	Right	CT-FFR for decision making	FFR adenosine
Zimmermann et al. [18]	2017	1	Case Report	FFR_CT_ *	Right	CT-FFR for decision making	FFR adenosine
Miki et al. [19]	2018	1	Case Report	FFR_CT_ *	Right	CT-FFR for decision making	FFR adenosine
Tahir et al. [20]	2018	1	Case Report	FFR_CT_ *	Left	CT-FFR for decision making	N/A
Pascoe et al. [21]	2019	1	Case Report	cFFR *	Right	CT-FFR for decision making	FFR adenosine
Tang et al. [22]	2020	94	Systematic Study	cFFR *	Right	Anatomical high-risk features correlation with CT-FFR	N/A
Adjedj et al. [23]	2021	54	Systematic Study	FFR_CT_ *	Both and Cx and single coronary arteries	CT-FFR in patients with an interarterial course versus other anomalous vessels	N/A
Ferrag et al. [24]	2021	62	Systematic Study	FFR_CT_ *	Both and Cx	CT-FFR in patients with and without high-risk features. Optimal cut-off to detect IMC	N/A
Medepalli et al. [25]	2021	1	Case Report	FFR_CT_ *	Right	CT-FFR for decision making	N/A
Bigler et al. [26]	2022	1	Case Report	cFFR *	Right	CT-FFR compared to invasively measured FFR	FFR adenosine and FFR dobutamine
Adjedj et al. [27]	2021	41	Systematic Study	QFR *	Right	QFR for outcome prediction in R-AAOCA	N/A
Rigatelli et al. [28]	2019	13	Systematic Study	ANSYS (CFD) **	Left	Comparison of WSS and vorticity magnitude of AAOCA with and without IMC	N/A
Cong et al. [29]	2020	26	Systematic Study	ANSYS (CFD) **	Right	Comparison of WSS and pressure between normal RCA and R-AAOCA	N/A
Rigatelli et al. [30]	2020	21	Systematic Study	ANSYS (CFD & FEA) **	Both	Virtual stenting of the IMC and post-procedural WSS and vorticity magnitude	N/A
Cong et al. [31]	2021	26	Systematic Study	ANSYS (FSI) **	Right	Comparison of WSS, volumetric flow and pressure between a normal RCA and R-AAOCA	N/A
Razavi et al. [32]	2021	6	Systematic Study	SimVascular (CFD) **	Both	Comparison of pre- and postoperative WSS and oscillatory shear index for different high-risk features	N/A
Chidyagwai et al. [33]	2022	13	Systematic Study	HARVEY (CFD) **	Both	Comparison of rest and stress conditions for AAOCA and then compared to normal coronaries	N/A
Jiang et al. [34]	2022	6	Systematic Study	SimVascular (FSI) **	Right	Comparison of FSI model of aortic root during dobutamine stress to invasively measured iFR	iFR dobutamine

* = *dedicated CFD* software for coronary arteries; ** = *general-purpose CFD* model approaches in AAOCA. All studies included in the final analysis. Abbreviations: AAOCA = anomalous aortic origin of a coronary artery, L-AAOCA = left anomalous aortic origin of the coronary artery, R-AAOCA = right anomalous aortic origin of the coronary artery, CFD = computational fluid dynamics, cFFR = CT-FFR from Siemens Healthineers, CT-FFR = computed tomography-derived fractional flow reserve, Cx = Circumflex artery, FFRCT = CT-FFR from Heartflow^®^, FEA = finite element analysis, FFR = fractional flow reserve, FSI = fluid–solid interaction, IMC = intramural course, iFR = instantaneous wave-free ratio, QFR = quantitative flow ratio from Medis^®^, RCA = right coronary artery, and WSS = wall shear stress.

**Table 2:** All studies using *dedicated* CFD models.

First Author	Software	Year	n (Patients)	n (IA)	n (R-AAOCA)	n (L-AAOCA)	CT-FFR All	CT-FFR Right	CT-FFR Left	FFR_adenosine_	FFR_dobutamine_
Pascoe et al. [21]	cFFR	2019	1	1	1	0		0.95		0.89	
Tang et al. [22]	cFFR	2020	94	94	1	0		0.94 (0.88–0.96)			
Bigler et al. [26]	cFFR	2022	1	1	1	0		0.79		0.77	0.72
Adjedj et al. [16]	FFR_CT_	2016	1	1	0	1			0.82		
Kawaji et al. [17]	FFR_CT_	2017	1	1	1	0		0.67		0.75	
Zimmermann et al. [18]	FFR_CT_	2017	1	1	1	0		0.5		0.53	
Miki et al. [19]	FFR_CT_	2018	1	1	1	0		0.77		0.65	
Tahir et al. [20]	FFR_CT_	2018	1	1	0	1			0.82		
Ferrag et al. [24]	FFR_CT_	2021	62	37	37	11	0.8 (0.74–0.88 IMC), 0.96 (0.93–0.98 not IMC)				
Medepalli et al. [25]	FFR_CT_	2021	1	1	1	0	0.75				
Adjedj et al. [16]	FFR_CT_	2021	54	33	31	2	0.90 ± 0.10	0.89 ± 0.20	0.85 ± 0.09		
Adjedj et al. [27]	QFR	2021	41	41	41	0	0.90 ± 0.10				

Abbreviations: AAOCA = anomalous aortic origin of a coronary artery, L-AAOCA = left anomalous aortic origin of the coronary artery, R-AAOCA = right anomalous aortic origin of the coronary artery, CT-FFR = computed tomography-derived fractional flow reserve, cFFR = CT-FFR from Siemens Healthineers, FFR_CT_ = CT-FFR from Heartflow^®^, IA = interarterial course, IMC = intramural course, FFR = fractional flow reserve, and QFR = quantitative flow ratio from Medis^®^.

**Table 3:** Studies with new CFD models compared by the typical steps of setting up a model. All studies with *general-purpose CFD* models compared by the different steps needed to create a CFD model. Abbreviations: L-AAOCA = anomalous aortic origin of a left coronary artery, R-AAOCA = anomalous aortic origin of a right coronary artery, CCTA = coronary computed tomography angiography, CMR = cardiovascular magnetic resonance imaging, FSI = fluid–solid interaction, iFR = instantaneous wave-free ratio, IMC = intramural course, LCA = left coronary artery, RCA = right coronary artery, and WSS = wall shear stress.

First Author	Number of Models Made	Imaging Modality and Segmentation	Mesh Quality	Newtonian/non-Newtonian, Blood Density [kg/m^3^] and Viscosity	Inlet Boundary Condition	Outlet Boundary Conditions	Wall Boundary Conditions	Steady State vs. Transient	Post Processing
Cong et al. [31]	26 (16 normal RCAs and 10 R-AAOCA)	CCTA and half-automatic segmentation with Materialize Mimics, aortic root, and coronaries	Mesh independence study performed, maximum face size 0.0008 m, 3 expansion layers	Newtonian, 1060, viscosity of 3.5 × 10^−^³ Pa s	Pulsatile flow matching human condition with Fourier series	Constant value with outflow pressure of Aorta to 56 Pa and coronary to 0 Pa	No slip, elastic with Young’s modulus of 5 MPa, and Poisson’s ratio of 0.45	Transient	Comparison of WSS, pressure, and volumetric flow over cardiac cycle in R-AAOCA compared to normal RCA
Rigatelli et al. [28]	13 L-AAOCA (6 intramural vs. 7 only interarterial)	CCTA and manual segmentation with OsiriX, postprocessed with Rhinoceros, aortic root, and coronaries	Ansys Meshing but no specifications	Non-Newtonian, 1060, Carreau	Diastolic pressure from stress tests of healthy athletes, constant inlet pressure	N/A	N/A	Steady	WSS and vorticity magnitude in patients with and without an IMC in rest and stress conditions
Cong et al. [29]	42 (16 normal RCA and 26 R-AAOCA)	CCTA and Mimics for segmentation, Geomagic Studio for optimizing geometry, aortic root, and coronaries	ICEM with mesh size between 0.06 and 1 mm, for fluid 5 mesh layers with 1.2 height ratio and 0.5 mm mesh size	Newtonian, 1060, -	Velocity inlet with tangential velocity of 1 m/s and normal velocity of 0 m/s	Aorta tangential pressure of 93 mmHg, LCA 81.83 mmHg, RCA 92.71 mmHg and normal pressure of 0	Vessel wall density 1150, Young’s modulus 5 MPa, Poisson ratio 0.45	Steady	Volumetric flow and pressure in normal RCA and R-AAOCA
Rigatelli et al. [30]	12 R-AAOCA and 9 L-AAOCA with IMC	CCTA and manual segmentation with OsiriX and postprocessed with Rhinoceros, aortic root, and coronaries	Ansys Meshing but no specifications	Non-Newtonian, 1060, Carreau	Pressure inlet with diastolic pressure from patient-specific stress test	N/A	N/A	Steady	WSS and vorticity magnitude before and after virtual stenting. Deformation analysis on geometries before and after
Chidyagwai et al. [33]	6 R-AAOCA, 2 L-AAOCA, 5 Controls	CCTA, Segmentation with Materialize Mimics, only coronaries	Mesh independence study showed convergence at 0.02 mm	Newtonian, 1060, -	Pulsatile Velocity profile at inlet, based on Doppler measurements. For exercise 3x higher cardiac output chosen	Lumped parameter model with microcirculation resistance, chosen to match clinical diastolic and systolic pressure.	Rigid walls, no slip condition,	Transient	WSS and oscillatory shear index in the intramural segment during rest and stress compared to normal anatomy
Razavi et al. [32]	3 R-AAOCA, 3 L-AAOCA (2 pre-unroofing and 2 post-unroofing), further virtual models with different acute take-off angles	CMR, Segmentation with SimVascular, aortic root, and coronaries	Mesh independence study until <5% change in results resulting in volumetric mesh of 3.5 × 10^6^elements	Newtonian,-, viscosity of 4cP	Inlet with volumetric flow derived from CMR for aorta, flow to each branch with Murray’s law and 4% of total cardiac output	Lumped parameter model with flow and resistance modelled to match 4% of total cardiac output and mean blood pressure	Aortic and coronary compliance in lumped parameter model to match measured blood pressure curve	Transient	WSS and oscillatory shear index pre- and post-unroofing
Jiang et al. [34]	6 R-AAOCA, 5 with an IMC	CCTA, segmentation performed in SimVascular, optimization in MeshMixer, aortic root, and coronaries with offset for aortic wall 1.7 mm and coronaries 0.9 mm then adjusted to match IMC	Mesh generated such as at least 2 elements for walls and 5 elements for fluid domain, mesh independence study performed	Newtonian, 1040, viscosity of 0.4 dynes/cm^2^	Neumann boundary condition to match aorta pressure waveform from iFR measurements at rest and stress (high frequency artefacts removed with fast Fourier transform)	Lumped parameter model to match cardiac output based on echocardiography, for stress increase 3×, resistance for aorta and coronaries based on heathy patients, capacitance 0.001cm^5^/dyne	Elastic wall for aorta and coronaries with E 1.5 MPa, poison ratio of 0.49, and density of 1.2 g/cm^3^	Transient FSI model	Comparison of CFD iFR during stress conditions compared to invasively measured iFR under dobutamine stress

## Data Availability

Not applicable.
